# A review of recent evidence on androgen abuse from interviews with users

**DOI:** 10.1097/MED.0000000000000834

**Published:** 2023-09-06

**Authors:** Bonnie Grant, Suks Minhas, Channa N. Jayasena

**Affiliations:** a Section of Investigative Medicine, Imperial College London, Commonwealth Building, Hammersmith Hospital; b Department of Urology, Charing Cross Hospital, London, UK

**Keywords:** anabolic–androgenic steroids, androgens, interviews, testosterone

## Abstract

**Purpose of review:**

Androgens (also known as anabolic–androgenic steroids; AAS) are increasingly being abused worldwide to enhance body physique or athletic performance. Qualitative studies including interviews provide a wider understanding of androgen abuse and focus specific support needs to this group. This narrative review summarizes recent studies (2021–2023) using interviews with individuals abusing androgens.

**Recent findings:**

Motivations for androgen abuse in men include desires to achieve certain physicality, enhance self-confidence and improve libido. The risks of androgen abuse are justified to achieve these outcomes and men may use other illicit substances as postcycle-therapy to lessen the risks. Some adverse effects may be more pronounced with certain substances. The therapeutic relationship with healthcare professionals is often described negatively by androgen abusers due to stigma and a perceived lack of knowledge. Both healthcare professionals and androgen abusers agree that development of guidelines are needed. Androgen abuse in women is rare however body dissatisfaction and desires for improve appearance and strength are motivators.

**Summary:**

Recent qualitative studies have helped further our understanding of men and women who abuse androgens, however the small number of recently published studies confirms there is still a paucity of evidence in the literature. Further research is needed to develop specific harm minimization strategies in those abusing androgens.

## INTRODUCTION

Image and performance enhancing drugs (IPEDs) are used worldwide with the aim of enhancing body physique or athletic performance. Androgens (also known as anabolic–androgenic steroids; AAS) are the most commonly used IPED [1,2]. Androgens are synthetic substances which mimic the effects of the natural hormone testosterone, needed in men and women for strength, muscle growth, mood and fertility.

Lifetime prevalence rates for androgen abuse in men have been estimated at 0.7–6.4%; however, these are likely to be underestimated as a result of nondisclosure by men abusing androgens [3–6]. Androgen abuse is illegal in many countries and therefore studies can prove difficult to undertake due to participant fears of retribution and criminal prosecution.

The use of qualitative methods such as interviews and focus groups has widely been used in addiction research [7]. By placing the participants perspective central to research, real-world narratives develop, which promotes a greater understanding of participant views and experiences alongside the influence of societal and environmental factors. Fear of stigma and the illicit nature of androgen abuse may discourage many users from participating in research. Interview based studies are a useful tool in reaching these ‘hidden’ groups by establishing trust and rapport [7]. By understanding the differing needs within this community, targeted harm minimization strategies can be developed.

This narrative review summarizes recent studies (2021–2023) involving interviews with individuals abusing androgens (Table 1). 

**Box 1:**
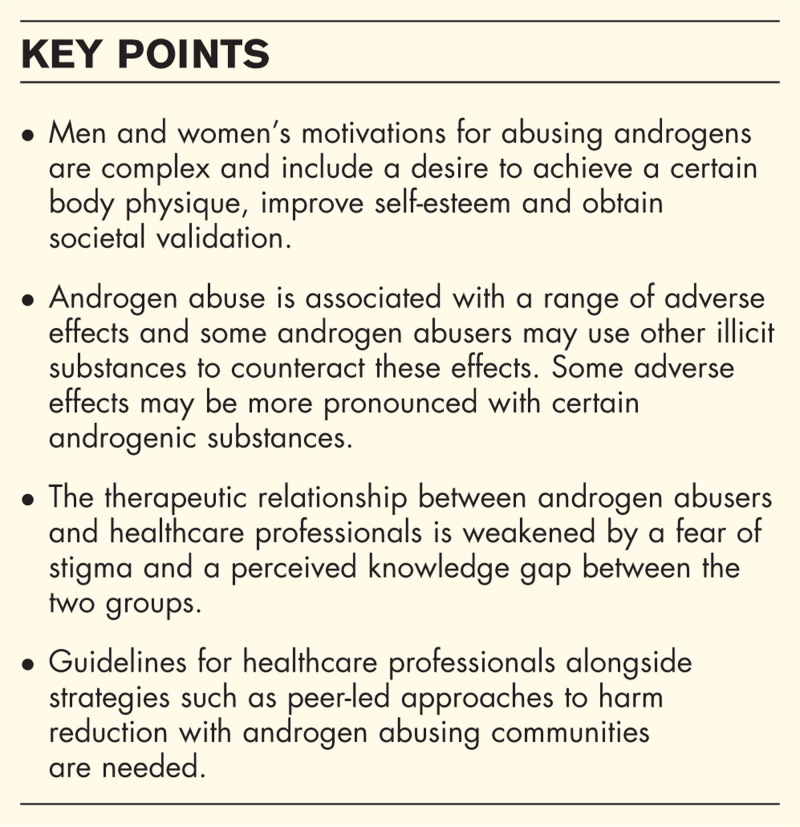
no caption available

**Table 1:** Summary of key findings of included studies using interview methodology

First author last name, year [Ref.]	Study design	Participants	Key findings
Börjesson, 2021 [9^▪^]	Interviews	Male Age 20–65 years Current (*n* = 6) or former (*n* = 6) abuse of androgens	• Androgen abuse helped achieve the ideal male physique, improve self-confidence, self-esteem and acceptance • A perfect self-image is fragile and subject to judgement from others in society
Harvey, 2022 [10^▪▪^]	Mixed-methods – questionnaire and interview	Questionnaire *n* = 133 Interviews *n* = 23 Male Aged 20–52 years	• Increased libido was a positive, although often unexpected effect of androgen abuse • Diminished libido upon cessation was a motivator to restart abuse • Loss of libido after androgen abuse was not felt to be taken seriously by medical professionals
Piatkowski, 2023 [17^▪▪^]	Interviews	Male (*n* = 9) and female (*n* = 7) Age 24–45 years Current or former abuse of androgens	• Trenbolone abuse was highly effective at gaining muscle and increasing energy however it also had the largest number of adverse effects • Trenbolone abuse was reported to cause increased aggression, emotional instability and cognitive deficits when compared with other androgenic substances
Piatkowski, 2021 [18^▪^]	Interviews	Male Age 16–30 years *n* = 12 Current or former abuse of androgens	• The extent of androgen abuse escalated in preparation for upcoming events • Party drug use was common in those also abusing androgens • Polysubstance use was reported by participants to increase aggression, violent behaviour and hospitalization
Ainsworth, 2022 [22^▪▪^]	Interviews	Androgen abuse group (*n* = 6) Male Physician group (*n* = 6)	Androgen abuse group • Fear of stigma and disclosing androgen abuse to healthcare professionals • Physicians lacked adequate knowledge to support their needs but hoped for future collaborative clinical interactions Physician group • Lack of knowledge, guidelines and resources was common • Discourse of their role toward those abusing androgens; some viewed themselves as educators while other were gatekeepers to services
Dunn, 2023 [23^▪^]	Interviews	General practitioners (GP) (*n* = 6)	• Knowledge was lacking and they were unable to adequately support those abusing androgens • Some were concerned that refusal to prescribe androgens would negatively impact on the relationship with their patients, while others were concerned about providing support to those engaging in illegal use
Piatkowski, 2022 [24^▪^]	Interviews	Androgen abuse group (*n* = 8) Male Mean age 27.87 years Service providers for IPED users’ group (*n* = 8)	• Mistrust toward healthcare professionals prevented those abusing androgens from seeking support from these traditional routes • Healthcare professionals reported a lack of knowledge and specific training, and this was a barrier to possible therapeutic relationships • Harm reduction services need to be developed further and may provide an opportunity for peer-led approaches
Havnes, 2021 [25^▪^]	Interviews	Female Age 19–46 years Current (*n* = 6) or former (*n* = 10) abuse of androgens	• Motivations for androgen abuse included desires to achieve an ideal body physique and improve mental health • Male androgen abusers were a source of trusted information • Menstrual cycle effects are used to monitor efficacy and safety of androgen abuse • Adverse effects such as voice and genital changes were negative and could inadvertently disclose androgen abuse
Börjesson, 2021 [27^▪▪^]	Interviews	Female Age 21–56 years *n* = 12 Current or former abuse of androgens	• Androgen abuse in women is viewed as a way to achieve body ideals • Body anxiety and dissatisfaction was common • Improved self-esteem was achieved by these bodily changes • The adverse masculinizing effects need to be counteracted to maintain femininity and keep androgen abuse hidden

## MOTIVATIONS FOR ANDROGEN ABUSE

Androgen abuse was predominantly used to enhance professional sporting endeavours and bodybuilding competitions in the 1970 and 1980s. Research over the past 20 years has reported a shift in motivations to a recreational use within the general population, aiming to achieve an aesthetically pleasing body physique of greater muscle mass and lean body fat, seen as the body ideal in modern society [8].

Börjesson *et al.* interviewed 12 men; half current androgen abusers and half previously abusing androgens [9^▪^]. The phenomenological analysis performed found that men within the study justified androgen abuse to achieve a certain physicality based on perceived masculinity ideals. This would involve implementing and maintaining strict self-discipline regimes. Men reported greater self-confidence and self-esteem as a result of achieving these set ideals and therefore an acceptance within social groups. One participant explained androgen abuse as “best antidepressant ever, best self-esteem and better self-confidence” [9^▪^]. This constant pressure to achieve goals, often intertwined with concealing androgen abuse is described as overwhelming with a fear of societal judgement and misunderstanding of their motivations and questioning their place in society.

A study by Harvey *et al.* reported libido to be a motivator in androgen abuse [10^▪▪^]. This mixed-methods study interviewed 23 participants out of 133 who had completed a questionnaire on experiences of androgen abuse. In line with the changing population of androgen abusers, only 6/23 of the participants described themselves as competitive athletes/bodybuilders. The desire to improve libido is not often reported as a primary motivator for starting androgen abuse; however, men interviewed did describe increased libido as a positive, albeit unintended effect. When stopping androgen abuse, decreased libido was a significant motivation for re-starting and for a number it became the primary motivation for ongoing use. However, increased libido was not always deemed a positive outcome with three participants interviewed reporting it to have a detrimental effect.

## ADVERSE EFFECTS OF ANDROGEN ABUSE

A number of studies have reported the associated adverse effects with the abuse of androgens including cardiac hypertrophy, stroke, gynaecomastia, infertility and hypogonadism post androgen abuse [11–14]. Despite knowledge of potential adverse effects, many men continue to abuse androgens and find ways to mitigate these effects. Men interviewed by Börjesson *et al.* explained that the potential risks associated with androgen abuse were justified to achieve the physical changes desired [9^▪^].

In an attempt to minimize the adverse effects of androgen abuse on the hypothalamic–pituitary–gonadal axis upon cessation, many men illicitly self-administer postcycle therapy (PCT). PCT often involves the use of at least one of; human chorionic gonadotropin (HCG), selective oestrogen receptor modulators (SERMs) or aromatase inhibitors (AI) with the overall aim to restore endogenous testosterone production more rapidly [15,16]. There is currently no evidence to support the use of PCT. Griffiths *et al.* interviewed 26 participants on their experience using PCT [15]. Participants described using PCT as a method to maintain their health and mitigate against adverse effects while also maintaining the muscle strength they had gained during training. Difficulty in accessing PCT was an emergent theme, with participants reporting obtaining androgens easier in comparison to PCT, resulting in continued abuse of androgens. Finally, many described low mood and anxiety when stopping androgen abuse with some improvements with PCT however others reported worsening mental health with PCT use.

Piatkowski *et al.* reported on users experience with the androgen trenbolone [17^▪▪^]. Trenbolone is an injectable androgen, derived from the nandrolone group and is a commonly used IPED due to its increased potency compared with testosterone. Participants interviewed in the study described trenbolone as a highly effective substance for increasing muscular development, but it was also reported to have more pronounced psychological effects compared with other androgens. Furthermore, some men reported psychological effects specific to trenbolone use such as aggression and cognitive deficit, which were not reported with other androgens. The current literature describing adverse effects with androgen abuse has grouped substances together, but it may be that differing adverse effects could be attributable to specific substances. If so, therapies could be targeted to ameliorate adverse effects specific to certain androgenic substances.

In a further study Piatkowski *et al.* interviewed 12 men on the extent of IPED use and polysubstance misuse [18^▪^]. Increasing IPED use was attributed to ease of accessibility, often through peers. Preparation for certain events (such as festivals or bodybuilding shows) was described as a motivator and the desire to maintain a certain aesthetic led to men to use or escalate IPED doses in order to achieve this. The men interviewed in this study reported aggression, violent behaviour and hospitalization in relation to combined IPED use and other substance misuse. Some of the men described other substance misuse as a way to relax and “blow off steam” after a strict regime of training including IPED use. Almost all men interviewed also used ecstasy (3,4-methylenedioxymethamphetamine: MDMA), often used as a party drug, implying that androgen abuse has become integral to a specific social lifestyle and culture for some men.

## RELATIONSHIP BETWEEN HEALTHCARE PROFESSIONALS AND ANDROGEN ABUSERS

IPED users have tended to describe their therapeutic relationship with healthcare professionals negatively. Physician knowledge is rated poorly by androgen abusers with a fear of stigma or judgement from healthcare professionals [19]. Harm reduction services are often used by individuals abusing androgens, however there is a considerable variability of service provision and infrastructure, reflecting the lack of guidance for professionals working within this field [20]. As a result, androgen abusers describe reluctance to seek traditional medical services and advice due to distrust and lack of treatment options and prefer to seek advice from peers [21].

Recent studies interviewing both androgen abusers and healthcare professionals has further evaluated this; Ainsworth *et al.* undertook semi-structured interviews with androgen abusers and physicians to evaluate each groups perception of the other [22^▪▪^]. Within the androgen abusing group the themes that emerged regarding physicians was a lack of knowledge, fear of stigma and disclosure of use but an overall want and need for collaboration with physicians in managing risks associated with androgen abuse. Importantly there was a feeling of power imbalance within the androgen abusing group, who felt that physicians were “service gatekeepers”, limiting access to services as a result of their own views on androgen abuse. Within the physician group, the themes had broad similarities with the perception that there was a lack of knowledge and guidelines but a motivation for their development. Many commented that they had no prior clinical exposure to men who were abusing androgens, and their own preconceptions could influence negatively on clinical interactions. There was also uncertainty within the physician group about their professional roles and responsibilities; some viewed themselves as educators and patient advocates while others saw themselves as gatekeepers to other services. In keeping with this, Dunn *et al.* reported that general practitioners were concerned that refusal to prescribe certain substances such as testosterone could negatively impact on their relationship with men abusing androgens, while others were concerned about providing support to those engaging in illegal use [23^▪^].

A similar study was published by Piatkowski *et al.* who interviewed eight male current or previous androgen abusers and eight service providers from either general practice clinics or harm reduction clinics [24^▪^]. Three themes emerged. The first was the need for a ‘Safe Space’ for users of IPEDs. This echoed previous findings, whereby the stigma felt by androgen abusers prevented them from seeking advice from traditional healthcare routes. This tied in with the second theme of low trust in healthcare professionals and a perceived lack of knowledge, particularly general practitioners, was a barrier to possible therapeutic relationships. This was reiterated by the healthcare professionals in the study who expressed concern over the lack of education, knowledge and specific training. This knowledge gap often resulted in androgen abusers seeking advice from peers and leading to potential isolation and spread of misinformation within the IPED community. Finally, there was an agreement between the groups that while harm reduction services are frequented by androgen abusers there is a need for them to develop beyond safe-injecting techniques. The authors commented that these services provide a unique opportunity for peer-led approaches to engage in harm reduction.

Overall, these studies suggest an ongoing, historical discourse between IPED users and healthcare professionals which continues to weaken the therapeutic relationship with androgen abusers seeking alternative medical advice and support as a substitute.

## WOMEN AND ANDROGEN ABUSE

Due to masculinizing effects, the majority of androgen abusers are male with little known about androgen abuse in women. Global lifetime prevalence rates of androgen abuse in women is estimated to be between 0.1–1.6% and research on androgen abuse in women is limited to small sample sizes [3,4,6]. In an attempt to minimize the virilizing effects of androgens, women have been reported to use fewer substances and at lower doses, compared to their male counterparts [25^▪^]. A higher proportion of women abusing androgen identify as competitive bodybuilders/weightlifters compared with men, however similarly to men, women often express body dissatisfaction and a desire for a leaner, more muscular physique as a motivator for androgen abuse [26].

For instance, Börjesson *et al.* interviewed 12 current or former female androgen abusers [27^▪▪^]. Women reported androgen abuse as a way to achieve a desired body physique, with accounts of eating disorders, body anxiety and dissatisfaction common amongst this group. Women interviewed often reported a history of bullying or negative comments of appearance in the past. Achieving these desired bodily changes and athletic achievements provides a form of external validation and approval from others, thereby improving self-esteem and confidence. Abusing androgens to achieve these effects was therefore justified.

Similar findings have been reported by Havnes *et al.* who performed semi-structured interviews for 16 women who reported current or prior androgen abuse [25^▪^]. Their analysis reported primary and secondary motivations for use. Primary motivations included desires for improved appearance and strength while secondary motivations included abusing androgens as a way to manage preexisting mental health problems including eating disorders. Abusing androgens also gave women a sense of self-protection and belonging within a community. The women interviewed in this study were almost exclusively introduced to abusing androgens by men. There was a strong reliance on men as the sole source of information on androgen abuse but many felt they were inadequately informed of the potential masculinizing adverse effects. Finally, women interviewed by Havnes *et al.* described positive and negative physical changes associated with androgen abuse. Changes in menstruation are used as a way to monitor the safety of androgen abuse; absence of menstruation is viewed as a positive and implies sufficient androgenic effect while return of menstruation assures the women of normal gonadal function and safe to resume androgen abuse. Voice and genital changes were seen as negative with concerns that these changes would expose their androgen abuse which is deemed unacceptable by society. Other studies have reported similar adverse effects of androgen abuse in women [28,29].

## STRENGTHS AND LIMITATIONS

The use of interviews with individuals abusing androgens allows for new information to emerge as interviews progress and for in-depth analysis of themes to occur. This may not be possible with other methods such as questionnaires which use more restrictive questioning [7,30].

A limitation of interview methodology is the time and expert knowledge required by investigators to complete them. This can result in small sample sizes, as evident in the studies reviewed here with the largest consisting of 23 interviews. As the studies discussed were voluntary, there is the potential to introduce bias. Participants who have had a notably positive or negative experience with androgen abuse may be more likely to consent to participation. Combined with small sample sizes, the conclusions drawn may not reflect the wider community.

## CONCLUSION

Recent qualitative studies have helped to further our understanding of the views and experiences of men and women who abuse androgens, however the small number of recently published studies confirms there is still a paucity of evidence in the literature. A wide range of motivations for androgen abuse has been explored beyond those for enhanced athletic performance. Motivations for androgen abuse between men and women show a number of similarities however more studies need to be done with female users. Interestingly, there may be a narrative developing of differing adverse effects between substances however the provenance of many of these substances obtained illicitly limits any conclusions. It is however clear that mistrust continues between androgen abusers and traditional healthcare professionals, resulting in a barrier to the therapeutic relationship, which could ultimately result in ongoing harms. Management guidelines for healthcare professionals alongside strategies such as peer-led approaches to harm reduction within androgen abusing communities need to be developed to minimize harm from androgen abuse.

## Acknowledgements

*ORCID: 0000-0002-2578-8223.*

### Financial support and sponsorship

*The Section of Endocrinology and Investigative Medicine is funded by grants from the MRC, NIHR and is supported by the NIHR Biomedical Research Centre Funding Scheme and the NIHR/Imperial Clinical Research Facility. The views expressed are those of the author(s) and not necessarily those of the NHS, the NIHR or the Department of Health. The following authors are also funded as follows: NIHR Post-Doctoral Fellowship (C.N.J.).*

### Conflicts of interest

*C.N.J. received investigator-led grants from Logixx Pharma Ltd.*

## REFERENCES AND RECOMMENDED READING

Papers of particular interest, published within the annual period of review, have been highlighted as:

▪ of special interest
▪▪ of outstanding interest
